# Supplementary material for: OpenMM 7: Rapid development of high performance algorithms for molecular dynamics
Source: PLoS Comput Biol. 2017 Jul 26;13(7):e1005659. doi: 10.1371/journal.pcbi.1005659 (PMC5549999; doi:10.1371/journal.pcbi.1005659)
Supplement: S1 Source Code — (ZIP) [file pcbi.1005659.s002.zip › OpenMM-7.0.1-Source/docs/api-c++/generated/OpenMM.HarmonicBondForce.html]

HarmonicBondForce — OpenMM @OPENMM\_MAJOR\_VERSION@.@OPENMM\_MINOR\_VERSION@ documentation


# HarmonicBondForce¶

*class* `OpenMM::``HarmonicBondForce`¶
:   This class implements an interaction between pairs of particles that varies harmonically with the distance between them. To use it, create a `HarmonicBondForce` object then call `addBond()` once for each bond. After a bond has been added, you can modify its force field parameters by calling `setBondParameters()`. This will have no effect on Contexts that already exist unless you call `updateParametersInContext()`.

    Methods

    |  |  |
    | --- | --- |
    | `HarmonicBondForce` | Create a `HarmonicBondForce`. |
    | `getNumBonds` | Get the number of harmonic bond stretch terms in the potential function |
    | `addBond` | Add a bond term to the force field. |
    | `getBondParameters` | Get the force field parameters for a bond term. |
    | `setBondParameters` | Set the force field parameters for a bond term. |
    | `updateParametersInContext` | Update the per-bond parameters in a `Context` to match those stored in this `Force` object. |
    | `usesPeriodicBoundaryConditions` | Returns whether or not this force makes use of periodic boundary conditions. |

    `HarmonicBondForce`()¶
    :   Create a `HarmonicBondForce`.

    int `getNumBonds`() *const*¶
    :   Get the number of harmonic bond stretch terms in the potential function

    int `addBond`(int *particle1*, int *particle2*, double *length*, double *k*)¶
    :   Add a bond term to the force field.

        |  |  |
        | --- | --- |
        | Parameters: |  |

        - **particle1** – the index of the first particle connected by the bond
        - **particle2** – the index of the second particle connected by the bond
        - **length** – the equilibrium length of the bond, measured in nm
        - **k** – the harmonic force constant for the bond, measured in kJ/mol/nm^2

        |  |  |
        | --- | --- |
        | Returns: | the index of the bond that was added |

    void `getBondParameters`(int *index*, int &*particle1*, int &*particle2*, double &*length*, double &*k*) *const*¶
    :   Get the force field parameters for a bond term.

        |  |  |
        | --- | --- |
        | Parameters: |  |

        - **index** – the index of the bond for which to get parameters
        - **particle1** – [out] the index of the first particle connected by the bond
        - **particle2** – [out] the index of the second particle connected by the bond
        - **length** – [out] the equilibrium length of the bond, measured in nm
        - **k** – [out] the harmonic force constant for the bond, measured in kJ/mol/nm^2

    void `setBondParameters`(int *index*, int *particle1*, int *particle2*, double *length*, double *k*)¶
    :   Set the force field parameters for a bond term.

        |  |  |
        | --- | --- |
        | Parameters: |  |

        - **index** – the index of the bond for which to set parameters
        - **particle1** – the index of the first particle connected by the bond
        - **particle2** – the index of the second particle connected by the bond
        - **length** – the equilibrium length of the bond, measured in nm
        - **k** – the harmonic force constant for the bond, measured in kJ/mol/nm^2

    void `updateParametersInContext`(Context &*context*)¶
    :   Update the per-bond parameters in a `Context` to match those stored in this `Force` object. This method provides an efficient method to update certain parameters in an existing `Context` without needing to reinitialize it. Simply call `setBondParameters()` to modify this object’s parameters, then call `updateParametersInContext()` to copy them over to the `Context`.

        The only information this method updates is the values of per-bond parameters. The set of particles involved in a bond cannot be changed, nor can new bonds be added.

    bool `usesPeriodicBoundaryConditions`() *const*¶
    :   Returns whether or not this force makes use of periodic boundary conditions.

        |  |  |
        | --- | --- |
        | Returns: | true if force uses PBC and false otherwise |

High performance molecular simulation on GPUs


### Navigation

- Core Objects
- Forces
- Integrators
- Extras

©2015, Stanford University and the Authors.
|
Powered by Sphinx 1.3.1
& Alabaster 0.7.7
|
Page source
